# Incidence and Characterisation of Methicillin-Resistant *Staphylococcus aureus* (MRSA) from Nasal Colonisation in Participants Attending a Cattle Veterinary Conference in the UK

**DOI:** 10.1371/journal.pone.0068463

**Published:** 2013-07-15

**Authors:** Gavin K. Paterson, Ewan M. Harrison, Emily F. Craven, Andreas Petersen, Anders Rhod Larsen, Matthew J. Ellington, M. Estée Török, Sharon J. Peacock, Julian Parkhill, Ruth N. Zadoks, Mark A. Holmes

**Affiliations:** 1 Department of Veterinary Medicine, University of Cambridge, Madingley Road, Cambridge, United Kingdom; 2 Department of Microbiological Surveillance and Research, Statens Serum Institut, Copenhagen, Denmark; 3 Health Protection Agency, Microbiology Services Division Cambridge, Level 6 Addenbrookes Hospital, Cambridge, United Kingdom; 4 Department of Medicine, University of Cambridge, Addenbrooke’s Hospital, Cambridge, United Kingdom; 5 Cambridge University Hospitals National Health Service Foundation Trust, Cambridge, United Kingdom; 6 The Wellcome Trust Sanger Institute, Wellcome Trust, Genome Campus, Cambridge, United Kingdom; 7 Moredun Research Institute, Bush Loan, Penicuik, United Kingdom; University Hospital Münster, Germany

## Abstract

We sought to determine the prevalence of nasal colonisation with methicillin-resistant *Staphylococcus aureus* among cattle veterinarians in the UK. There was particular interest in examining the frequency of colonisation with MRSA harbouring *mecC*, as strains with this *mecA* homologue were originally identified in bovine milk and may represent a zoonotic risk to those in contact with dairy livestock. Three hundred and seven delegates at the British Cattle Veterinarian Association (BCVA) Congress 2011 in Southport, UK were screening for nasal colonisation with MRSA. Isolates were characterised by whole genome sequencing and antimicrobial susceptibility testing. Eight out of three hundred and seven delegates (2.6%) were positive for nasal colonisation with MRSA. All strains were positive for *mecA* and none possessed *mecC*. The time since a delegate’s last visit to a farm was significantly shorter in the MRSA-positive group than in MRSA-negative counterparts. BCVA delegates have an increased risk of MRSA colonisation compared to the general population but their frequency of colonisation is lower than that reported from other types of veterinarian conference, and from that seen in human healthcare workers. The results indicate that recent visitation to a farm is a risk factor for MRSA colonisation and that *mecC*-MRSA are rare among BCVA delegates (<1% based on sample size). Contact with livestock, including dairy cattle, may still be a risk factor for human colonisation with *mecC*-MRSA but occurs at a rate below the lower limit of detection available in this study.

## Introduction

*Staphylococcus aureus* is an important opportunistic pathogen associated with nosocomial and community-acquired infections in people, and is responsible for disease in animals where it is most economically significant as a cause of bovine mastitis [1], [2].

Methicillin-resistant *S. aureus* (MRSA) have acquired one of a number of staphylococcal cassette chromosome *mec* elements (SCC*mec*) [3], carrying a gene (*mecA*) encoding a penicillin binding protein (PBP 2a) with low affinity for β-lactam antibiotics [4]. Since 2005 there have been a number of reports suggesting that the rate of carriage of MRSA is higher in people living or working on pig farms than in the wider community due to zoonotic acquisition of MRSA, primarily belonging to the clonal complex (CC) 398 lineage. Although initially associated with pigs, subsequent reports indicate that other domestic animal species are also affected including veal calves [5], dairy cattle [6], poultry [7] and horses [8].

In 2011 we described a previously unreported divergent *mecA* homologue [9]. Genome sequencing revealed a *mecA* homologue (*mec*A_LGA251_, now designated *mecC* [10]) within a new SCC*mec* element (type XI). A search of human *S. aureus* isolates from national collections in the UK and Denmark identified MRSA with *mecC*, originating from human carriage and disease as well as further bovine isolates from England. Significantly, isolates with identical sequence types and *spa*-types were found in human and bovine isolates, suggesting transmission between the two host populations. Strengthening this supposition of interspecies transmission, work in Denmark has identified *mecC* MRSA human isolates from two different farms that are identical by sequence type, *spa* type, MLVA profile, and PFGE pattern to isolates from cattle or sheep on those farms [11]. Subsequent genome sequencing revealed that these human and animal isolates differed by only a few single nucleotide polymorphisms across the core genome, substantiating that transmission events had occurred between host species [12]. Little is known of the epidemiology of *mecC* MRSA but its prevalence appears to be increasing in Denmark [11] and it has also been reported from the Republic of Ireland [13], France [14], Norway [15], Germany [16], Switzerland [17], Holland [18] Sweden [19] and Belgium [20]. In addition to humans, cattle and sheep, *mecC* MRSA have also been isolated from domestic dog, domestic cat, guinea pig, common seal, chaffinch, rabbit and brown rat [15], [20], [21]. A *mecC* homologue, *mecC1*, has also been described in *Staphylococcus xylosus* from bovine mastitis, within a putative ancestral SCC*mec*XI [22]. Importantly, *mecC* MRSA produce a negative result in *mecA*-based PCR and PBP2a slide agglutination assays and as such have the potential to be misdiagnosed as methicillin-sensitive *S*. *aureus*.

Although there is some evidence that there is transmission between people and animals of MRSA encoding *mecC*, further evidence is required to test this hypothesis, and to obtain evidence concerning the direction of this transmission. If there is transmission from cows to people then cattle veterinarians might be expected to be at a high risk of carriage of the new MRSA through their occupational exposure. A survey of participants at a cattle veterinary conference held in the UK in November 2011 was performed in order to determine the carriage rate of MRSA and to examine the epidemiology of any MRSA recovered.

## Materials and Methods

### Study Setting and Participants

The annual British Cattle Veterinarian Association (BCVA) Congress 2011 was held at the Southport Theatre and Convention Centre, Merseyside, UK from 24th to 26th November 2011. BCVA is the cattle specialist division of the British Veterinary Association. Approximately 400 delegates, including veterinary students and exhibitors attended. All volunteers completed a questionnaire asking for the approximate time since last contact with livestock or a visit to a farm. The country in which they worked and the first part of their postcode (UK based delegates only) were also recorded.

### Sample Collection and Processing

Sterile Ames media transport swabs (Medical Wire, Corsham, UK) were used to sample both anterior nares. The swabs were subcultured within 48 hours onto MRSA Brilliance Agar 2 (Oxoid, Baskingstoke, UK) and incubated for 24 hours at 35°C. We have isolated *mecC* MRSA from humans using this approach previously (data not shown) and using a collection of twelve known *mecC* MRSA from humans and dairy cattle have found that all grew well on this agar. Single colonies of methicillin-resistant isolates including potential MRSA were taken for further characterisation. Plates with no growth were further incubated to a total of 48 hours at 35°C yielded no further putative MRSA. The presence of *femB*, *mecA* and *mecC* was tested by multiplex PCR as previously described [23]. Three non-*S*. *aureus* methicillin-resistant isolates were identified to the species level using a MALDI Biotyper (Bruker Daltonic GmBH, Bremen, Germany) MALDI-TOF mass spectrometer. Total cellular protein extractions were prepared in formic acid and analysed according to the manufacturer’s recommendations. Swabbing of the anterior nares of the experimenters found them to be negative for MRSA at the time of the conference.

### Antimicrobial Resistance Testing

Antimicrobial susceptibility testing was performed by disc diffusion (Oxoid, Basingtoke, UK) according to the European Committee on Antimicrobial Susceptibility Testing (EUCAST) methodology (www.eucast.org, (v 2.1, 7 Feb 2012)) for 12 antimicrobial agents: penicillin, cefoxitin, norfloxacin, erythromycin, clindamycin, kanamycin, tetracycline, linezolid, fusidic acid, rifampicin, trimethoprim/sulfamethoxazole and mupirocin. All susceptibility results were interpreted according to EUCAST guidelines with the exception of trimethoprim/sulfamethoxazole, for which interpretation was made according to CLSI guidelines. In addition the MIC was determined for cefoxitin and oxacillin by microbroth dilution performed as described by EUCAST using Mueller Hinton BBL II broth (Becton Dickinson, Heidelberg, Germany) and a final inoculum of 5×10^5^ CFU. *S. aureus* ATCC 29213 was used for quality control.

### Genome Sequencing and Analysis

Genomic DNA was extracted from overnight cultures by MasterPure™ Gram Positive DNA Purification Kit (Cambio, Dry Drayton, UK) and sequenced by HiSeq 2000 (Illumina Inc., Little Chesterfield, UK). Multi-locus sequence types and SCC*mec* types were derived from the genome sequences and antimicrobial resistance determinants identified by BLAST analysis [24].

### Ethics Statement

The study protocol was submitted for consideration for ethical review to the National Research Ethics Service (East of England), which reported that formal ethical approval was not required for this study because the subjects were healthy individuals in a non-healthcare environment, non-invasive sampling was used, and no human tissues were being collected. Delegates who volunteered to be swabbed were provided with an information sheet about the study and provided written informed consent for participation in the study.

### Statistics

Statistical analysis was performed using SPSS v20 (IBM Corporation). The threshold for statistical significance was an alpha error of 0.05. The Mann-Witney U Test was used to compare the time since subjects were last on a farm for MRSA positive and negative subjects as this data was not normally distributed.

## Results and Discussion

### MRSA Carriage Amongst Study Participants

**Figure 1:**
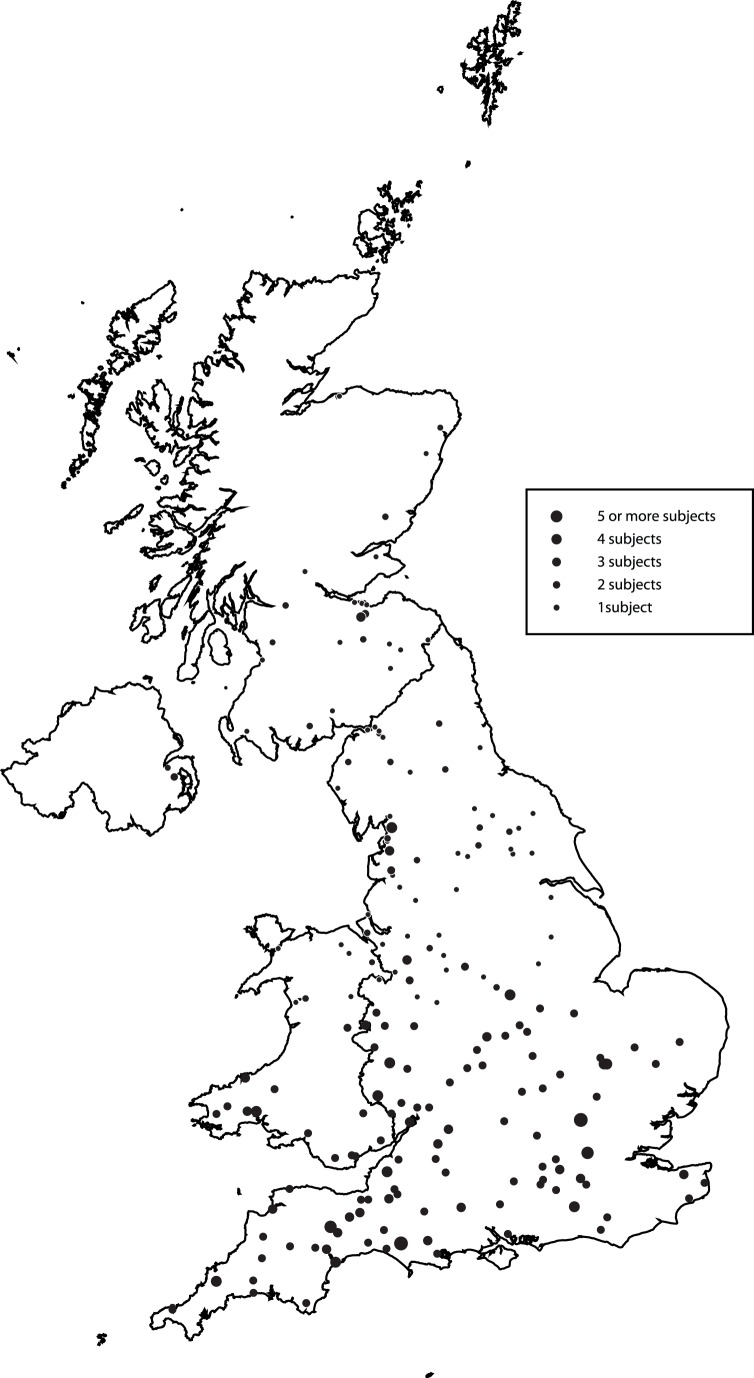
A map showing the approximate locations of the primary workplace of the UK subjects who participated in the MRSA carriage survey.

From the 307 swabs taken, eight produced growth characteristic of MRSA on MRSA Brilliance 2 plates, giving an overall carriage prevalence of 2.6% (95% CI 1.1–4.1%). The approximate geographical location of the primary work address reported by UK subjects is shown in Figure 1. Unsurprisingly, these locations reflect the general distribution of dairy farming in the UK (cattle farms in the UK are concentrated in western regions). The primary place of work of nine participants was outside the UK.

The MRSA carriage rate in the general population has been reported to be in the range of 0.8–1.3% [25], [26], [27], [28]. The observed MRSA colonisation rate in delegates at the BCVA 2011 conference of 2.6% was higher than the rate expected in the general population, but lower than the 4.6% observed in human healthcare workers [29] as well as the carriage rates obtained from screening attendees at other veterinary conferences, Table 1. For instance, a survey of Dutch veterinarians and veterinary students found an overall carriage rate of 4% [30], and a survey of British small animal veterinarians found a rate of 17.9% [31]. Participants at veterinary conferences provide a convenient sample of veterinary practitioners. Although a proportion of people attending these conferences may have little direct contact with animals (e.g. company sales representatives, students or academics, industry and government veterinarians), the majority of participants are likely to have frequent, direct interactions with animals under their care. The median time between swabbing and last visit to a farm was shorter in the MRSA-positive group (median = 1 day, range 1–14 days) than in the MRSA-negative group (median = 3 days, range 0.1–365 days) although not statistically significant using a Mann-Whitney U Test (p = 0.08). The Australian study of a number of different conferences provides a useful comparison of veterinarians working with different species, and reported a 4.7% MRSA carriage rate in veterinarians working with cattle, although this figure has wide confidence intervals (0.57–15.81) due to the small denominator (n = 43) [32]. The carriage rate in small animal veterinarians was comparable (4.9%, n = 430), while the carriage rate in equine veterinarians was considerably greater (11.9%, n = 202) [33]. There are however differences in methodology between studies which may affect rates of isolation and it should be noted that the lack of a broth enrichment step may have reduced the sensitivity of MRSA detection in this survey.

**Table 1:** A summary of the results from previous MRSA carriage surveys undertaken at veterinary or animal health conferences.

Conference	Country	Year	MRSA rate	No. of subjects	Reference
Cattle	UK	2011	2.6%	308	This study
Pig Health	Denmark	2006/7	12.5%	272	[64]
Multiple	Denmark	2006/7	1.9%	574	[65]
ACVIM	USA	2005	6.5%	417	[66]
AAEP	USA	2006	10.1%	257	[67]
ACVS	USA	2008	17.3%	341	[68]
Multiple	Australia	2009	5.8%	771	[33]
Dermatology	Italy	2010	1.6%	128	[69]

ACVIM, The American College of Veterinary Internal Medicine; AAEP, American Association of Equine practitioners; ACVS, American College of Veterinary Surgeons.

The occupational nature of the risk association with MRSA carriage suggested in this study is strengthened by the association between a subject’s recent presence on a farm and them testing positive for MRSA. It is reasonable to suggest that participants at the congress who had recently visited a farm were more likely to be actively engaged in clinical work on farms and therefore have increased exposure to livestock, and/or, that colonisation by MRSA is rapidly lost after occupational exposure. In support of these suggestions, there is evidence that carriage rates of livestock-associated CC398 MRSA in veal calf farmers are associated with intensity of animal contact and rapidly decrease in the absence of contact with livestock [34].

While the increased rate of MRSA carriage in human health workers might be explained by greater exposure to MRSA through close contact with patients with MRSA and associated fomites. This is less likely to be the situation in veterinary medicine as there is no evidence of high carriage rates of MRSA in UK livestock. The occupational risk of MRSA acquisition and carriage by veterinarians may be associated with working in environments where antibiotics are present, which might offer a selection advantage to colonising bacteria that are resistant. In this regard even low, sub-inhibitory concentrations may be sufficient to provide a selective advantage to resistant strains [35]. Alternatively or additionally, many SCC*mec* elements contain genes that provide resistance to other bactericidal agents (e.g. the arsenical resistance gene in type XI *SCCmec* [36]) and these could explain an increased survival of MRSA in veterinary environments.

### Characterisation of Methicillin-resistant Staphylococci from Study Participants

All eight MRSA isolates where *femB* and *mecA* gene positive by PCR but negative for *mecC*. Genome sequencing confirmed each isolate as being *mecA*-positive MRSA, and selected genotypic and phenotypic characteristics are shown in Table 2. Antimicrobial susceptibilities were compared with the presence/absence of known resistance determinants or mutations (the so-called ‘resistome’) [37]. As described previously for ST22 MRSA [37], antimicrobial phenotypes and genotypes in our study show concordance, further supporting that whole-genome sequencing may in future have a role in informing therapy and represents a powerful tool for the discovery of new drug-resistance mechanisms [9].

**Table 2:** Characteristics of the eight MRSA isolates found in the survey of conference participants.

Strain	Sequence Type	Clonal Cluster	*spa* type	SCC*mec* type	Region of workplaceof subject	Ox MIC	Fox MIC	Additional resistance^1^	Resistance determinates^2^
BCVA7	ST22	CC22	t032	Type IVa-ACME	Somerset, England	>128	128	NOR	GyrA S84L
BCVA16	ST2274	CC22	t879	Type IVa	Cornwall, England	>128	64	NOR ERY CLI	GyrA S84L *ermC ermT*
BCVA92	ST398	CC398	t011	Type IVa	Continental Europe	64	32	KAN ERY CLI TET	GyrA S84L *tetM tetL aacA-aphD dfrK ermC ermT*
BCVA124	ST2014	CC8	t008	Type IVh	Aberdeenshire, Scotland	128	64	TET	*tetK*
BCVA182	ST22	CC22	t032	Type IVh	Devon, England	128	64	NOR ERY CLI	GyrA S84L *ermC ermT*
BCVA191	ST22	CC22	t032	Type IVh	Denbighshire, Wales	>128	64	NOR	GyrA S84L
BCVA289	ST8	CC8	t008	Type IVa	Devon, England	32	32	NOR ERY	GyrA S84L*msrA*, *mphC*
BCVA296	ST59	CC59	t216	Type IVa	Gloucestershire, England	64	64		

1 Susceptibilities tested: linezolid, rifampicin, kanamycin, norfloxacin, erythromycin, clindamycin, fusidic acid, tetracycline, trimethoprim/sulfamethoxazole and mupirocin.

2 Genome screened for *tetM* M21136, *tetK* NC_017331_REGION: 69118.70497, *msrSA* AB013298_487.1953, *mphC* NC_017351 REGION: 53170.54069, *ileS* AJJR01000036 REGION: 742.3816, *fusC* HE980450 REGION: 10019.10657, *ermA* NC_002745 _N315, *dfrG* AB205645 REGION: 1013.1510, AB568461 *Staphylococcus aureus aacA*-*aphD* gene for bifunctional AAC/APH, *gyrA* CP000046_COL, *rpoB* X64172 REGION: 1222.4770, *tetL* JN970906 REGION: 8454.9830, *ermC* NC007792 REGION: 7865.8599 *ermT* NC017673 REGION: 1795197.1795946.

There were three methicillin-resistant non-*S*. *aureus* isolates that grew on MRSA Brilliance 2 agar, which were identified as *Staphylococcus haemolyticus* by MALDI-TOF. By PCR these were negative for *femB* and *mecC* but positive for conventional *mecA* and each showed resistance to several other antibiotics, Table 3. These displayed relatively high MICs to oxacillin and cefoxitin and resistance to multiple other antibiotics. Little data are available on carriage rates of *S*. *haemolyticus* but it is a nosocomial pathogen characterised by resistance to multiple antimicrobial agents [38], [39].

**Table 3:** Characteristics of the three *S*. *haemolyticus* isolates found in the survey of conference participants.

Strain	Oxacillin MIC (mg/l)	Cefoxitin MIC (mg/l)	Additional resistances^1^
BCVA70	>128	64	KAN, NOR, ERY, CLI, TET,
BCVA233	>128	>256	KAN, NOR, ERY, CLI, FUS, TET, SXT
BCVA257	>128	256	KAN, NOR, ERY, CLI, FUS, TET, SXT

1 Susceptibilities tested: linezolid, rifampicin, kanamycin, norfloxacin, erythromycin, clindamycin, fusidic acid, tetracycline, trimethoprim/sulfamethoxazole and mupirocin.

The ST398 MRSA isolated in this study came from a delegate from continental Europe where ST398 is the predominant lineage of LA-MRSA. In the UK MRSA ST398 is apparently rare with only two published reports to date [40] [41], including our recent description of MRSA ST398 isolated from bulk tank milk from five dispersed UK dairy farms which may represent an emerging problem in the UK [41]. Heterogeneity is seen within the ST398 population with human and livestock-associated lineages differentiated by the presence or absence of specific virulence factors and resistance genes [42], [43]. The absence of the *sak*, *chp*, and *scn* genes and the presence of *tet*(M) in BCVA198 indicates that it belongs to the livestock-associated lineage.

Four of the eight MRSA isolates belonged to CC22. BCVA 7, 182 and 191 were ST22 while BCVA16 was a novel single locus variant in *arcC*, ST2274. CC22 is a diverse and widespread lineage common in many countries [44], [45], including in England where it was responsible for >75% of MRSA bacteraemia between 2001–7 [46]. In addition to its importance in humans, this lineage has also been isolated from a range of animals: cats, dogs, horses, bats, turtles, pet birds, pigs and goats [31], [47], [48], [49], [50], [51]. While it has yet to be reported from cattle, this host promiscuity may contribute to it being the most common MRSA lineage in our survey of veterinarians. Phylogenetic analysis of core genome single nucleotide polymorphisms showed that all four isolates mapped into the large ST22-A2 cluster identified by Holden *et al.* [45] (data not shown) suggesting they are closely related to hospital associated ST22 isolates.

Strains BCVA289 (ST8) and BCVA124 (ST2014) belonged to CC8. BCVA289 appears to belong to the most prominent MRSA strain in North America [44]: USA300, an important source of community-acquired MRSA. The characteristic USA300-like features of BCVA289 were being ST8, *spa*-type t008, SCC*mec*-IVa, Panton-Valentine leukocidin (PVL)-positive, arginine catabolic mobile element-positive, and having more than six AT repeats within the SACOL005 locus (a feature used in combination with PVL for PCR identification of USA300 strains [52]). The isolation of a USA300 strain from nasal colonisation of a UK delegate (based in the South West of England) was unexpected as USA300 strains remain rare in the UK compared to the USA [53]. The second CC8 isolate, BCVA124 did not belong to the USA300 clone, it was; ST2014 (a SLV of ST8), *spa*-type t008, was negative for PVL and *arcA*, and encoded five AT repeats within SACOL005 instead of the 6 or more associated with USA300 [52].

ST8 MSSA have been found in bovine milk in Japan [54] and ST8 MRSA found in bovine milk in Turkey [55]. ST8-MRSA-*SCCmec*IV, t008, appears to favour equine colonization in veterinary clinical settings and have been known to cause infections in horses. Simultaneous colonization of veterinary personnel attending horses has also been reported [56], [57]. A German study from 2007 identified a ST8 MRSA in a case of bovine mastitis [58], and a recent report from Switzerland has identified MSSA ST8 as an emerging lineage responsible for cases of bovine mastitis in that country, with the suggestion that the pattern of host specificity is changing [59]. The final MRSA isolate BCVA296 was from the CA-MRSA lineage ST59. Representatives of this lineage have been isolated in the UK but particular strains, both PVL positive and negative, are more prevalent in Australia, Taiwan and the US as a source of community-acquired MRSA infection [44], [60]. A study of MRSA obtained from livestock environments and livestock workers in Taiwan identified ST59 as the predominant lineage [61] and a MSSA ST59 obtained from bovine milk is listed in the *S. aureus* MLST database (saureus.mlst.net). ST59 MRSA have been found in carriage studies of cats and dogs in the Japan and Taiwan [62], [63].

The aim of this study was to look for evidence of transmission between cow and humans of MRSA harbouring the newly described *mecA* homologue *mecC*. The failure to detect any *mecC* MRSA isolates provides evidence to indicate that the carriage rate of these MRSA strains in UK cattle veterinarians is likely to be less than 1%. A calculation of the binomial exact confidence intervals reveals that our sample size of 307 would have had a 95% probability of finding at least 1 positive result if the prevalence had been 1%. However, the prevalence of MRSA among UK cattle, both *mecA* and *mecC* MRSA, is not yet known so it is unclear how likely occupation exposure is for cattle veterinarians. The origins and epidemiology of MRSA *mecC*, including the risk factors associated with its acquisition remain unclear. Prevalence studies in the general population and in dairy cattle are currently underway in the UK. Recent contact with dairy cattle may yet be an important risk factor for human colonisation with *mecC*-MRSA but occurs at rates below the lower limit of detection in this study.
